# Doubly Phosphorylated Peptide Vaccines to Protect Transgenic P301S Mice against Alzheimer’s Disease Like Tau Aggregation

**DOI:** 10.3390/vaccines2030601

**Published:** 2014-07-29

**Authors:** Monique Richter, Agneta Mewes, Manuela Fritsch, Ute Krügel, Ralf Hoffmann, David Singer

**Affiliations:** 1 Institute of Bioanalytical Chemistry, Faculty of Chemistry and Mineralogy, Universität Leipzig, Deutscher Platz 5, Leipzig 04103, Germany; E-Mails: monique_richter@gmx.de (M.R.); agneta.mewes@bbz.uni-leipzig.de (A.M.); manuelafritsch@hotmail.de (M.F.); ralf.hoffmann@bbz.uni-leipzig.de (R.H.); 2 Center for Biotechnology and Biomedicine (BBZ), Universität Leipzig, Deutscher Platz 5, Leipzig 04103, Germany; 3 Rudolf Boehm Institute for Pharmacology and Toxicology, Universität Leipzig, Leipzig 04107, Germany; E-Mail: Ute.Kruegel@medizin.uni-leipzig.de

**Keywords:** Alzheimer’s disease, immunization, peptide vaccine, phospho-tau, transgenic mouse model, P301S mice

## Abstract

Intracellular neurofibrillary tangles and extracellular senile plaques are potential targets for active and passive immunotherapies. In this study we used the transgenic mouse model P301S for active immunizations with peptide vaccines composed of a double phosphorylated tau neoepitope (pSer202/pThr205, pThr212/pSer214, pThr231/pSer235) and an immunomodulatory T cell epitope from the tetanus toxin or tuberculosis antigen Ag85B. Importantly, the designed vaccine combining Alzheimer’s disease (AD) specific B cell epitopes with foreign (bacterial) T cell epitopes induced fast immune responses with high IgG_1_ titers after prophylactic immunization that subsequently decreased over the observation period. The effectiveness of the immunization was surveyed by evaluating the animal behavior, as well as the pathology in the brain by biochemical and histochemical techniques. Immunized mice clearly lived longer with reduced paralysis than placebo-treated mice. Additionally, they performed significantly better in rotarod and beam walk tests at the age of 20 weeks, indicating that the disease development was slowed down. Forty-eight weeks old vaccinated mice passed the beam walk test significantly better than control animals, which together with the increased survival rates undoubtedly prove the treatment effect. In conclusion, the data provide strong evidence that active immune therapies can reduce toxic effects of deposits formed in AD.

## 1. Introduction

Alzheimer’s disease (AD) is the fifth leading cause of death for people aging 65 years and older [1]. Thus, many efforts have been devoted to reveal the pathogenesis of AD and to develop disease-modifying therapies. AD is biochemically and histochemically characterized by two pathological protein aggregates: extracellular senile plaques (SP) and neurofibrillary tangles (NFT). SP contain amyloid β (Aβ) peptide 1–40/42, which is generated by secretases from the amyloid precursor protein (APP) [2,3], whereas NFT are intracellular deposits of hyperphosphorylated tau [4,5,6,7]. These disease-related aggregates are initiated in neurons, astrocytes and oligodendrocytes by alternative enzymatic degradation pathways (APP) or pathological hyperphosphorylation (tau protein). These aggregates may affect the structure and dynamic regulation of the cytoskeleton subsequently disturbing axonal transport resulting in a loss of synaptic integrity, degeneration of neurons, and finally a loss of cognitive functions.

Current AD therapeutic strategies aim to reduce or ideally to prevent or even reverse the formation of toxic Aβ and tau species using small molecules (e.g., aggregation inhibitors) or active and passive immunization strategies [8,9]. Immunotherapies should provide prophylactic protection or treatment options before the first clinical symptoms appear to keep cognitive deficits at a low level. Thus immunogens and small molecules are preferably administered after early diagnosis of the first symptoms of mild cognitive impairment (MCI) well before AD [10,11]. Recently several promising Aβ-immunotherapies have advanced from preclinical to clinical phases [12]. In contrast, tau immunotherapy is still in early development stages and, to the best of our knowledge, has not advanced to clinical trials, although the NFT burden can be reduced by both active and passive phosphorylation-dependent immunotherapy strategies [13,14,15]. Even more important, the cognitive abilities improved together with the diminished tau pathology in immunized animals [13,16,17].

Currently, substances for active (immunogenic peptides) and passive (humanized anti-Aβ or anti-phospho-tau monoclonal antibodies) immunization strategies are under development. While active immunization needs some time to initiate an immune response and depends on each individual, it has the potential to induce long lasting protection after a few vaccinations. Passive immunization provides an immediate and dose-dependent effect, but only for a short time period [8].

Vaccination typically relies on bacterial or viral particles, glycoproteins or short proteins and peptides, of which peptide-based vaccines have been successfully developed against infectious diseases (e.g., influenza A [18]) and different types of cancer (e.g., breast cancer [19]). However, clinical trials to treat neurodegenerative diseases like AD with peptide vaccines have failed so far [20,21].

Peptide immunogens are typically administered after conjugation to carrier proteins, such as keyhole limpet hemocyanin (KLH), ovalbumin (OVA) or bovine serum albumin (BSA). A serious side effect of carrier proteins represents the partial activation of a cytotoxic immune response (Th1) [22,23]. Such cytotoxic immune responses can be prevented by using short peptides containing only the desired epitope (≤20 residues) or multiple antigen peptides (MAPs), which carry two to 16 copies of an epitope on a branched lysine core, as successfully applied for influenza A [18]. Such peptides in combination with a Th2 supporting adjuvant were also used in first preclinical tau- and Aβ-related immunization studies [13,24].

Active immunization with MAPs requires adjuvants to achieve high antibody titers. As Freund’s adjuvant induces a strong pro-inflammatory immune response [25,26], aluminum salts are preferred due to anti-inflammatory immune stimulation [27]. In a previous study, we could overcome their major weakness of a low stimulatory effect by linking short, disease-specific phospho-tau epitopes to immune-stimulating T cell epitopes from *Clostridium tetani* (tetanus toxin, TT_582–599_) and *Mycobacterium tuberculosis* (Ag85B_241–255_) administered with aluminum hydroxide [28].

The aim of the present study was to develop an active prophylactic and/or therapeutic immunization strategy to reduce the NFT burden in transgenic P301S mice by targeting hyperphosphorylated tau protein with high antibody titers to postpone disease symptoms, increase the life span, and improve the health status of aged mice. Therefore, double phosphorylated tau neoepitopes (pSer202/pThr205, pThr212/pSer214, pThr231/pSer235), were chosen representing an early (pThr231/pSer235), an intermediate (pThr212/pSer214) and a late (pSer202/pThr205) phosphorylation event in AD [29,30].

## 2. Experimental

### 2.1. Solid Phase Peptide Synthesis

Peptides were synthesized using fluorenylmethoxycarbonyl/*tert-*butyl chemistry (Fmoc/^t^Bu) on polystyrene-based Rink amide (MBHA) resin (0.65 mmol/g, MultiSynTech GmbH, Witten, Germany). Protected amino acids (MultiSynTech GmbH) were coupled in eight molar excess using diisopropyl carbodiimide in the presence of 1-hydroxy-benzotriazole (DIC/HOBt) either on the Syro2000 multiple peptide synthesizer (MultiSynTech GmbH) or the microwave-assisted peptide synthesizer Liberty (CEM GmbH, Kamp-Lintford, Germany) in a 250 µmol scale.

### 2.2. Synthesis of Phospho-Tau Peptide Vaccines

Phosphorylated tau peptides Tau_199–208_[pS202/pT205], Tau_209–217_[pT212/pS214] and Tau_229–237_[pT231/pS235] were synthesized automatically on MBHA resin (250 µmol). Positions to be phosphorylated were incorporated with unprotected side chains, *i.e.*, Fmoc-Ser-OH and Fmoc-Thr-OH [31]. After synthesis of the B cell epitope and the linker sequence GPSL, the resin was divided into 125 µmol portions followed by coupling of either one of the T cell epitopes TT_582–599_ or Ag85B_241–255_ (Table 1, [28]). All six peptides were phosphorylated using amidite chemistry [31]. In brief, free hydroxyl groups were phosphitylated with dibenzyl-*N*,*N*-diisopropyl-phosphoramidite (15 eq) in the presence of 1H-tetrazole in a mixture of acetonitrile and dimethylformamide (DMF, 40 eq, 0,45 mol/L) at room temperature (RT) for 90 min and then repeated once with fresh reagents for 16 h. After washing, the phosphitylated peptides were oxidized twice with *tert*-butyl hydroperoxide (*^t^*BuOOH, 100 eq) in decane (RT, 90 min).

**Table 1:** Origin and sequences of T cell and B cell epitopes used to immunize transgenic P301S mice. * pS, pT, and hMAPT denote phospho-serine, phospho-threonine, and human microtubule-associated protein tau, respectively.

T cell epitope	Origin	Sequence *
TT	Tetanus toxin 582–599 of *Clostridium tetani*	VDDALINSTKIYSYFPSV
TBC	Ag85B 241–255 of *Mycobacterium tuberculosis*	QDAYNAGGGHNAVFD
**B cell epitope**		
Tau_199–208_[pS202/pT205]	hMAPT	SPG**pS**PG**pT**PGS
Tau_209–217_[pT212/pS214]	hMAPT	RSR**pT**P**pS**LPT
Tau_229–237_[pT231/pS235]	hMAPT	VR**pT**PPK**pS**PS

### 2.3. Peptide Cleavage, Purification and Analysis

Peptidyl resins were washed with DMF and dichloromethane (DCM), air dried and cleaved with 5% (v:v) water, 4% (v:v) thioanisol, 4% (v:v) m-cresol, and 2% (v:v) ethanedithiol in trifluoroacetic acid (TFA, 2 h, RT). Peptides were precipitated with ice cold diethyl ether, washed three times, air dried and stored at 4 °C. All peptide immunogens were purified on an Äkta HPLC System (Amersham Bioscience GmbH, Freiburg, Germany) using a Jupiter C_18_-column (21.2 mm × 250 mm, 15 µm particle, 300 Å pore size, Phenomenex Inc., Torrance, USA) and a gradient slope of 1% acetonitrile per minute in the presence of 0.1% (v:v) TFA as ion-pair reagent. The flow rate was 10 mL/min and UV absorbance was recorded at 220 nm. The purity of the peptides was judged by RP-HPLC using a Jupiter C_18_-column (2.0 mm × 150 mm, 3 µm particle, 300 Å pore size) and their masses were confirmed by matrix-assisted laser desorption/ionization time-of-flight mass spectrometry (MALDI-TOF-MS; 4700 proteomic analyzer; Applied Biosystems GmbH, Darmstadt, Germany) operated in positive ion-mode using α-cyano-4-hydroxy-cinnamic acid as matrix (Bruker Daltonics GmbH, Bremen, Germany). Alternatively, peptide purity was confirmed by liquid chromatography electrospray ionization mass spectrometry (QSTAR pulsar, ESI-QqTOF-MS, Applied Biosystems) using an aqueous acetonitrile gradient in the presence of 0.1% (v:v) formic acid.

### 2.4. Animals

B6C3F1 (Harlan Lab, Eystrup, Germany) female mice were purchased at the age of 4 to 6 weeks. Male P301S mice (B6;C3-Tg(Prnp-MAPT*P301S)PS19Vle/J) were obtained from Charles River (Erkrath, Germany). The transgenic construct contained the 412 residue long 4R/1N isoform of human MAPT with mutation P301S. The mutant was expressed under the control of the mouse prion protein (Prnp) promoter at five-fold higher levels than endogenous mouse MAPT. On average hyperphosphorylation and deposition of the overexpressed tau is detectable in six-month-old mice and mimics AD-like tau pathology. First behavioral symptoms are visible by three month of age with limb retraction and clasping when lifted by the tail followed by limb weakness that finally progresses to paralysis associated with a hunched back posture at the age of seven to ten months [32]. After mating B6C3F1 female mice with male transgenic P301S mice the presence of the transgene in hemizygous offspring’s was identified by PCR with genomic DNA extracted from ear or tail biopsies using the primer pair 5'-GGGGACACGTCTCCACGGGCATCTCAGCAATGTCTCC-3' and 5'-TCCCCCAGCCTAGACCACGAGAAT-3'. All mice were housed at 12 h light/dark cycles with unlimited excess to water and diet food (ssniff Spezialdiäten GmbH, Soest, Germany). The study was approved by the local authorities (Landesdirektion Leipzig, license number TVV 14/09) following the guidelines of the German Animal Welfare Act. All efforts were made to reduce the number of animals and to minimize animal suffering.

### 2.5. Immunization

**Figure 1:**
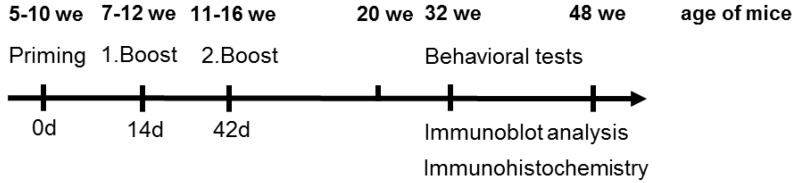
Time scale of the applied immunization protocol and behavioral tests, as well as the time points of immunoblot and immunohistochemical analysis, we = weeks.

Female mice were immunized with freshly prepared vaccine formulations. Peptides were dissolved in sterile Tris buffer (10 mmol/L, pH 7.4, 1 g/L), mixed (1:1, v:v) with Alu-GelS as adjuvant (Serva Electrophoresis, Heidelberg, Germany) and allowed to absorb onto the aluminum particles overnight at 4 °C on a tube rotator [13]. Mice were primed the next day subcutaneously at multiple sites followed by two intraperitoneal boosts 14 and 42 days afterwards (Figure 1). The immune response was evaluated by blood samples that were taken submandibular seven to ten days after each immunization and thereafter every four weeks for the next eight months. Red blood cells were removed by centrifugation (6700× *g*, 90 s) and the supernatant containing the serum was stored at −20 °C.

In total 160 transgenic female P301S mice (40 animals per group) were primed and boosted with 100 μg of peptide vaccine each time using a 1:1 (v:v) mixture of two peptides containing the same B cell epitope, *i.e.*, Tau_199–208_[pS202/pT205], Tau_209–217_[pT212/pS214], or Tau_229–237_[pT231/pS235], but only one of the two different T cell epitopes TBC and TT [13,16,33]. Control mice received an equal volume of a 1:1 (v:v) mixture of adjuvant and 10 mmol/L Tris buffer.

### 2.6. Enzyme-Linked Immunosorbent Assay (ELISA)

Specific antibody titers were measured by ELISA using terminally elongated Cys-Tau_196–211_[pS202/pT205], Cys-Tau_206–220_[pT212/pS214], Cys-Tau_226–240_[pT231/pS235] peptides without the T cell epitopes. Antisera were first screened for total IgG titers (0.04 µg/mL, goat-anti-mouse-(γFc)-POD, Dianova, Hamburg, Germany). Subsequently, a randomly chosen subset (*n* = 12) of sera with OD values above 1 in a dilution of 1:100 at the total IgG determination were screened for specific IgG_1_, IgG_2a_, IgG_2b_, and IgG_2c_ titers with the help of corresponding specific secondary antibodies (0.25 µg/mL, Southern Biotech, Birmingham, AL, USA) using an established ELISA procedure and a cut off value for titer determination of 0.1 AU [34]. In brief, a 96-well MaxiSorp immunoplate (Nunc brand, Thermo Fisher Scientific, Langenselbold, Germany) was coated with peptide antigens (2 µg/well for Tau_196–211_, Tau_206–220_, Tau_226–240_) dissolved in deionized water and dried at 37 °C overnight. Wells were blocked with blocking buffer (200 µL, 10 mmol/L sodium phosphate buffer, pH 7.4, 0.3 mol/L sodium chloride, and 0.05% (v:v) Tween^®^20, 0.5% (w:v) casein) for 1 h, washed three times with washing buffer (300 µL, 10 mol/L sodium phosphate buffer, pH 7.4, 0.3 mol/L sodium chloride, 0.05% (v:v) Tween^®^20), and incubated with diluted murine serum (ten-fold dilution series from 1:100 to 1:100,000) for 1 h at RT. The supernatant was discarded, plates were washed, and the secondary horseradish peroxidase-conjugated goat anti-mouse IgGγFc (IgG_1/2a/2b/2c_) was added at a dilution of 1:10,000 (1:4000) in washing buffer for 1 h. After washing, 1-Step-Ultra-TMB (Pierce Biotechnology, Rockford, IL, USA) was added (100 µL/well) and incubated (15 min, darkness). The reaction was stopped by addition of sulfuric acid (100 µL/well, 0.5 mol/L) and the absorption was measured at 450 nm (620 nm reference).

### 2.7. Behavioral Characterization

The health status of mice was monitored prior to each test session. The behavior of transgenic P301S (20 mice per immunization and placebo group) and age-matched non-transgenic mice (C3H/BL6, 10 animals) was investigated at 20, 32, and 48 weeks of age (Figure 1) using three different tests, as described below.

#### 2.7.1. Wire Hang Test

Mice were placed on a wire mesh (10 cm × 15 cm, 30 cm height) that was then inverted and waved gently, so that the mouse is forced to grip the wire. The latency to fall was recorded, with a 60 s cut-off time [35].

#### 2.7.2. Accelerating Rotarod Test

Motor coordination and locomotor activity were tested using an accelerating rotarod with four lanes (RotaRod 3375, TSE Systems, Bad Homburg, Germany). At an age of about 20 weeks, mice were habituated for the first time to the rod (30 mm diameter, 85 mm width) for 60 s at a constant speed of 4 rotations per min (rpm) for three trials. At training and experimental days the speed was accelerated from four to forty rpm over a period of 300 s. The fall down was registered by a light beam and the latency was recorded with TSE RotaRod Software [36]. Four trials with an inter trial interval of at least 15 min were performed on three consecutive days. Day one and two were defined as training sessions whereas the experiment was performed on day three. The average latency of each mouse to fall in all four trials was calculated and statistically evaluated.

#### 2.7.3. Beam Walk Test

Motor coordination and balance were tested by walking along a 1 m long beam. Mice were placed onto a start platform 50 cm above a padded surface and were trained to traverse the beam towards their home cage. Mice were trained twice on a beam with 23.5 mm diameter in the morning before tested twice using a beam of 15.5 mm diameter in the afternoon. The time to traverse the beam and the number of foot slips were recorded. Cut-off time was 60 s [37].

### 2.8. Primary Monoclonal Anti-phospho Tau Antibodies (mAb)

For immunohistochemistry and immunoblot analysis the following antibodies from Thermo Scientific (Langenselbold, Germany) were used: mouse anti-human PHF-tau IgG_1κ_ monoclonal antibody, clone AT8 (Tau[pS202/pT205]), mouse anti-human PHF-tau IgG_1κ_ monoclonal antibody, clone AT100 (Tau[pT212/pS214]) and mouse anti-human PHF-tau IgG_1κ_ monoclonal antibody, clone AT180 (Tau[pT231]) [38,39,40,41].

### 2.9. Immunohistochemistry

Coronal brain slices of 32 (*n* = 3–6) and 48 weeks (*n* = 5–9) old P301S mice were analyzed by immunohistochemistry (Figure 1). Animals were euthanized with carbon dioxide and transcardially perfused with phosphate-buffered saline (PBS, 10 mmol/L phosphate, 0.15 mol/L sodium chloride, pH 7.4) containing heparin (0.1%, w:v) followed by paraformaldehyde (4%, w:v). Brains were removed, post-fixed for 12 h, and stored in 30% sucrose containing sodium azide (0.1%, w:v). Coronal sections (30 μm) were cut from frozen brains on a sliding microtome (Leica Mikrosysteme Vertrieb GmbH, Wetzlar, Germany) and stored in sodium phosphate buffer (0.1 mol/L, pH 7.4) containing sodium azide (0.025%, w:v). The monoclonal antibodies AT8 (0.4 µg/mL) and AT100 (0.8 µg/mL) (Thermo Scientific, Langenselbold, Germany) were used to detect phosphorylated tau protein [38,39,40,41]. Prior to staining, slices were washed in sodium phosphate buffer (0.1 mol/L, pH 7.4, 10 min) and three times in Tris buffered saline (TBS, 0.1 mol/L Tris, 0.15 mol/L sodium chloride, pH 7.4, 10 min each). After inactivation of endogenous peroxidase with 1% (v:v) hydrogen peroxide in 60% (v:v) aqueous methanol (1 h, RT), brain sections were washed three times with TBS (10 min) and blocked with 5% (v:v) normal donkey serum in TBS (NDS, Dianova GmbH, Hamburg, Germany) containing 0.3% (v:v) Triton X-100 (30 min). Subsequently, tissue slices were incubated with the primary antibody dissolved in blocking buffer overnight at 4 °C with gentle shaking. After washing in TBS, brain slices were incubated with biotinylated donkey anti-mouse IgG (1:200, Vector Laboratories, CA, USA) as secondary antibody in 2% (w:v) bovine serum albumin/TBS (1 h, RT). For indirect detection of the immune complex ExtrAvidin^®^-peroxidase (Sigma Aldrich, 1:1000, St. Louis, MO, USA) and 3,3'-diaminobenzidine (DAB) were used for color development. All DAB-stained slices were counterstained by hematoxylin and mounted onto microscope slides in Entellan (Merck KGaA, Darmstadt, Germany). Digital images of areas of interest (hippocampal formation, bregma −1.70 to −2.18, [42]) were recorded with an Axioplan2 microscope with ×2.5, ×4.0, ×10, and ×20 objectives and a color video camera (Carl Zeiss AG, Göttingen, Germany). Quantitative image analysis was done with the imaging software ImageJ [43] with the plugin particle counter/cell counter (Kurt De Vos, University of Sheffield, Academic Neurology, Sheffield, UK). The evaluated areas (CA1, CA2/3/4, and DG) and the pathological structures counted are represented in Figure S1.

### 2.10. Preparation of Brain Homogenates

P301S mice at the age of 32 and 48 weeks (Figure 1) were euthanized with carbon dioxide and transcardially perfused with buffer containing sodium fluoride (0.11 mol/L), sodium pyrophosphate dibasic (10 mmol/L, pH 7.4), protease inhibitor mix M (1%, (v:v), Serva Electrophoresis, Heidelberg, Germany) and phosphatase inhibitor mix II (1%, (v:v), Serva Electrophoresis, Heidelberg, Germany). Brains were removed from the skull, snap frozen in liquid nitrogen, and stored at −80 °C. Afterwards the whole brains were homogenized on ice using a mixture of urea (7 mol/L), thiourea (2 mol/L), dithiothreitol (50 mmol/L), 4% (w:v) CHAPS and protease and phosphatase inhibitors (1%, v:v) [44,45] using a dounce homogenizer (0.2 g brain per mL). Insoluble parts were removed by centrifugation (15,000× *g*, 30 min, 4 °C) and the supernatants were stored at −80 °C. Protein concentrations were determined by Bradford assay.

### 2.11. Relative Quantification of Phospho-Tau

Supernatants of brain homogenates were diluted in sample buffer (62.5 mmol/L Tris-HCL, pH 6.8, 20% (w:v) glycerol, 2% (w:v) SDS, 5% (v:v) 2-mercaptoethanol, 0.025% (w:v) bromophenol blue to obtain a final protein concentration of 1 g/L. Samples (120 µL) were spiked with trypsin inhibitor (30 µL, 1.5 g/L, Trypsin Inhibitor Glycine max, Sigma Aldrich, Munich, Germany) resulting in a final protein concentration of 0.8 g/L and a final trypsin inhibitor concentration of 0.3 g/L. Denatured (5 min, 95 °C) samples (10 µL) were loaded in triplicates on a Mini-PROTEAN^®^ TGX^TM^ precast stain-free gel (12% T, 1.0 mm thick, Bio-Rad Laboratories GmbH, Munich, Germany) and separated in parallel in a Mini Protean Tetra Electrophoresis System (Bio-Rad Laboratories GmbH, Munich, Germany) for 25 min at 300 V (electrode buffer: 25 mmol/L Tris buffer, pH 8.3, 192 mmol/L glycerol, 0.1% (w:v) SDS). Tryptophan depending fluorescence was induced by UV light (5 min) immediately after electrophoresis and then recorded for 3 s (excitation 285 nm, emission 480 nm, Fusion FX7, PEQLAB Biotechnology GmbH, Erlangen, Germany).

### 2.12. Immunoblot Analysis

Proteins were electro-transferred from activated TGX gels onto PVDF-membranes (pore size 0.45 μm, Millipore GmbH, Schwalbach, Germany) in a semi dry transfer cell (25 V, 90 min, Bio-Rad) and detected on membranes (0.3 s) and in gels (3 s) after UV light-induced tryptophan-depending fluorescence. Membranes were blocked with nonfat dry milk powder (5%) in T-TBS (1.5 h, 0.1% Tween^®^20, 25 mmol/L Tris, pH 7.5, 150 mmol/L sodium chloride) and incubated with the mAbs AT8 (0.2 mg/L), AT100 (2.0 mg/L), AT180 (0.2 mg/L) and Tau5 (0.66 μg/L, Dianova GmbH, Hamburg, Germany) in blocking buffer (overnight, 4 °C). Membranes were washed three times with blocking buffer incubated with the secondary antibody in blocking buffer (1 h, 0.04 mg/L goat anti-mouse-(γFc)-POD, Dianova, Hamburg, Germany) and washed twice with T-TBS and TBS for 10 min each. Bands were visualized with a mixture of solutions A and B (1:1 (v:v), 0.1 mL/cm^2^) provided with the ECL Advance Western blotting detection kit (GE Healthcare Europe GmbH, Munich, Germany for mAbs AT8, AT100 and AT180, 5 min) or with the chemiluminescence reagent for HRP from Serva Electrophoresis (Heidelberg, Germany for mAb Tau5 1:1000 diluted with additional 30% (v:v) H_2_O_2_, 2 min). The free image software ImageJ was used for quantitative images analysis [43]. The total protein content was measured after blotting by tryptophan dependent fluorescence revealing an average variation of only 3.1% to 10.1% within one day and 3.2% to 10.9% between several days, which allows a reliable downstream statistical analysis. This was also confirmed by the band intensities of external standard. Signal variation for the spiked trypsin inhibitor was between 3.4% and 9.5% within one day and 2.9% to 10.9% among several days verifying the technical reproducibility. Densitometrical analyses of total tau and phospho-tau on the immunoblot membranes resulted in gray values of typically 3000 to 5000 for bands stained with mAb Tau5, ~20,000 for mAbs AT8 and AT180 and a wide range from 1000 to 20,000 for mAb AT100. These values were then normalized to the gray values obtained for the total protein content (approximately 500 to 1000). Brain homogenates of mice that showed less band intensities with mAb AT100, but a staining for the other three anti-tau antibodies were judged with a minimal value of 0.28 for the ratio of mAb AT100 to total protein and included in quantification. Bands of brain homogenates that were saturated with mAbs AT8 and AT180 were judged with a maximal value of 13.3 for the ratio of mAb AT8 to total protein and with a maximal value of 17.5 for the ratio of mAb AT180 to total protein. Samples displaying none of the expected four mAb band intensities were excluded for further analyses and considered as non-transgenic. All relevant densitometrical values were normalized to the total protein content of each sample. The percentage of the hyperphosphorylated tau was calculated relative to the total tau quantities and analyzed statistically comparing different immunization groups.

### 2.13. Statistical Data Analysis

Data were analyzed with the software GraphPad Prism [46] first testing parametric distribution with the normality test D’Agostino and Pearson omnibus. Three or more parameter analysis relied on one-way analysis of variance or Kruskal Wallis test followed by Dunn’s or Tukey’s multiple comparison method as *post hoc* analysis. Two-Factor analysis relied on two-way analysis of variance with Bonferroni as *post hoc* test. Planned comparisons for improvement of tau pathology and behavioral impairments were done with one-tailed student’s *t*-test or one-tailed Mann-Whitney test. Life span studies were analyzed using the Kaplan-Meier survival curve and log-rank (Mantel-Cox) test and the log-rank test for trend. The significance level for all statistical analyses was 0.05.

## 3. Results

### 3.1. Design and Peptide Synthesis of Immunogens

**Figure 2:**
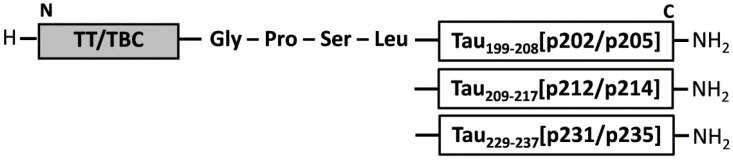
Schematic representation of the peptide vaccine design combining AD specific doubly phosphorylated epitopes of the tau protein (B cell epitope) via a tetrapeptide to foreign T cell epitopes to stimulate a non-inflammatory immune response.

All six peptides (Figure 2, Table 1) were synthesized in two steps connecting any of the three studied tau sequences (B cell epitope) via the GPSL linker sequence to either of the two T cell epitopes [28,47] with purities of 85 to 95% according to the peak areas of the reversed phase chromatograms. The post-synthetic global phosphorylation of positions Ser202/Thr205, Thr212/Ser214, and Thr231/Ser235 was very efficient yielding dominantly the desired double phosphorylated tau peptide, contaminated only with small amounts of single (TBC-Tau_199–208_[pS202/pT205] and TT-Tau_229–237_[pT231/pS235]) or triple phosphorylated peptides (TBC-Tau_199–208_[pS202/pT205] and TT-Tau_199–208_[pS202/pT205]). The yields ranged from 9.8% to 22.8% (Table 2). Further information regarding the peptide design and a detailed evaluation of their immunological properties with respect to specificity and cross reactivity can be found elsewhere [28].

**Table 2:** Analytical characteristics of the synthesized peptide vaccines. Retention times (t_R_) were obtained by analytical RP-HPLC.

B cell epitope	T cell epitope	t_R_ [min]	MM_calc_/MM_obs_	Yield [%]	Purity [%]
**Tau_199–208_[pS202/pT205]**	TT	31.5	3368.6/3368.7	15.4	90
	TBC	15.3	2858.2/2858.1	13.8	90
**Tau_209–217_[pT212/pS214]**	TT	26.5	3539.8/3539.7	21.8	95
	TBC	18.8	3029.4/3029.4	22.8	85
**Tau_229–237_[pT231/pS235]**	TT	21.8	3493.8/3493.8	10.4	>95
	TBC	26.9	2983.4/2983.6	9.8	95

### 3.2. Specific Total IgG Titers

**Figure 3:**
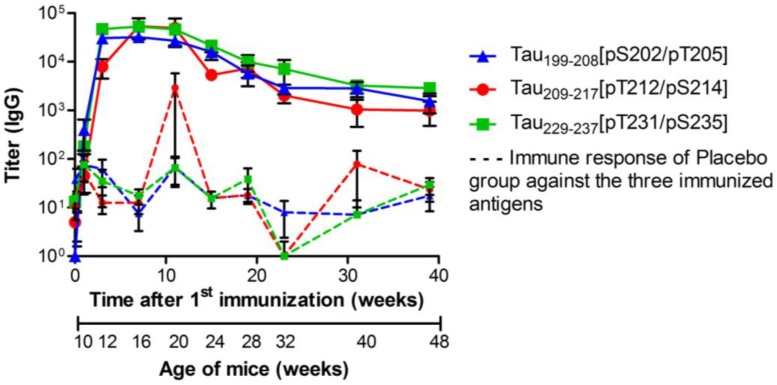
Specific total IgG titers of P301S mice immunized with Tau_199–208_[pS202/pT205] (blue triangle 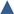, *n* = 39), Tau_209-217_[pT212/pS214] (red circle 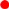, *n* = 39) or Tau_229–237_[pT231/pS235] (green square 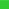, *n* = 36). Animals were immunized and boosted twice, two and six weeks after. Sera were collected one week before (background) and again 1, 3, 7, 11, 15, 19, 23, 31, and 39 weeks after the first vaccination (day 0). Solid lines represent the IgG titers of vaccinated mice; dashed lines represent the unspecific immune response against the three antigens, which was detected in sera of placebo treated mice (*n* = 36). Shown are geometric means of IgG titers with the standard error of the mean (SEM).

All three doubly phosphorylated tau sequences induced strong and fast immune responses in 62.5% (Tau_199–208_[pS202/pT205]), 77.8% (Tau_209–217_[pT212/pS214]) and 100% (Tau_22__9–237_[pT231/pS235]) of P301S mice, reaching similar specific total IgG titers of approximately 50,000 after two injections within three weeks (Figure 3, Table S1, Table S2, Table S3). The specific total IgG titers did not further increase after the second boost, but remained stable until week 20 without any further vaccination. In the following months, the titer dropped slowly to approximately 2500. Cross-reactivities towards the T cell epitopes and non-immunized phospho-tau epitopes were below 1000 (data not shown).

### 3.3. Specific IgG_1_/IgG_2a_/IgG_2b_/IgG2_c_ Titers

**Figure 4:**
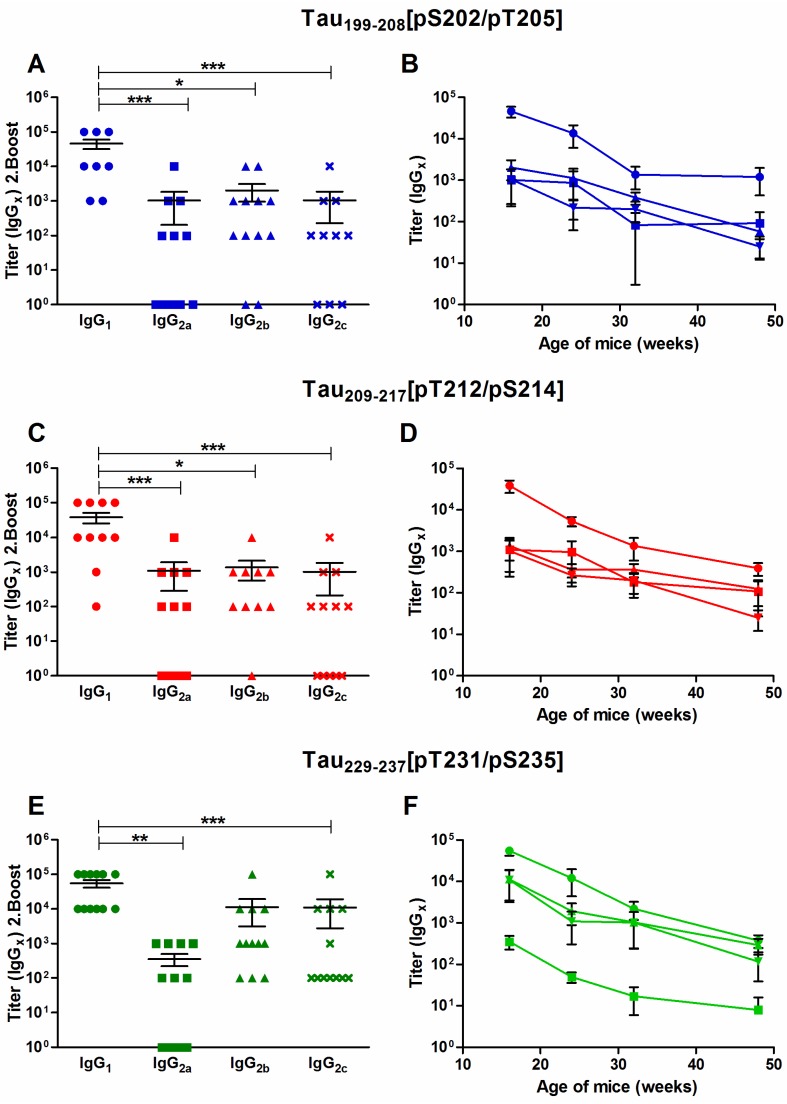
Specific titers of IgG_1_ (circles), IgG_2a_ (squares), IgG_2b_ (triangles) and IgG_2c_ (crosses) in sera samples collected one week after the second boost from P301S mice immunized with Tau_199–208_[pS202/pT205] (*n* = 12, (**A**)), Tau_209–217_[pT212/pS214] (*n* = 12, (**C**)), Tau_229–237_[pT231/pS235] (*n* = 12, (**E**)). Subsequent time course of IgG subtype titers at different time points until the end of the immunization study (**B**,**D**,**F**). Statistical significance is indicated by asterisks (*, *p* < 0.05; **, *p* < 0.01; ***, *p* < 0.001).

Detailed subtyping of a randomly chosen subset of 12 animals of each immunization group after the second boost (12- to 17-weeks-old P301S mice), showed IgG_1_ mean titers of 46,000 for Tau_199–208_[pS202/pT205], 38,500 for Tau_209–217_[pT212/pS214] and 55,000 for Tau_229–237_[pT231/pS235] (Figure 4, left column, Table S4, S5 and S6). Six of the 12 mice immunized with Tau_199–208_[pS202/pT205] and five of the 12 mice immunized with Tau_209–217_[pT212/pS214] and Tau_229–237_[pT231/pS235] showed no IgG_2a_ titers at all, whereas the other 50% showed IgG_2a_, IgG_2b_, and IgG_2c_ titers of approximately 1500 for mice immunized with Tau_199–208_[pS202/pT205] and Tau_209–217_[pT212/pS214]. Immunization with Tau_229–237_[pT231/pS235] initiated IgG_2a_ titers below 500. IgG_2b_ and IgG_2c_ titers were detected in all sera of about 11,000. During the following month IgG_1_, IgG_2a_, IgG_2b_ and IgG_2c_ mean titers decreased continuously (Figure 4, right column) similar to the total specific IgG titers (Figure 3). It should be noted although IgG_2a_ and IgG_2c _originate from the same IgG locus; both were analyzed because of the mixed BL6/C3H genetic background of the animals.

### 3.4. Tau Phosphorylation Degrees in Brain Homogenates

**Figure 5:**
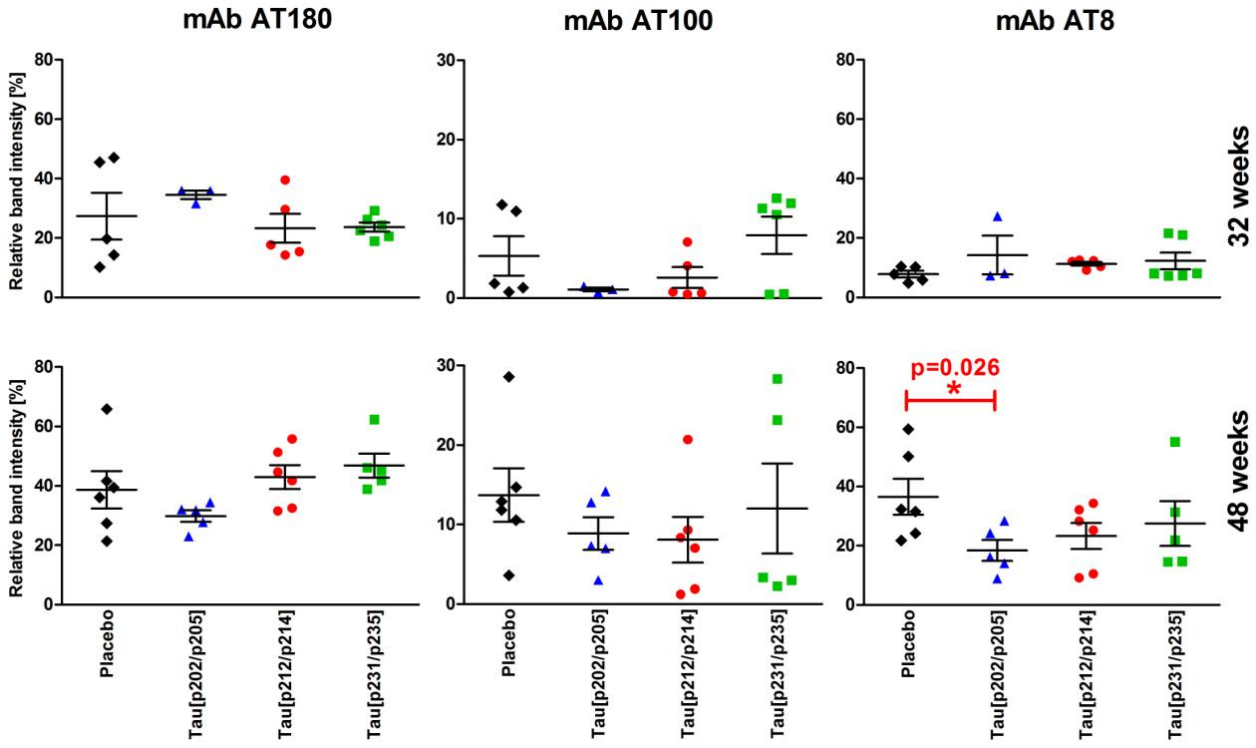
Normalized phospho-tau contents in brain homogenates of placebo-treated P301S mice (*n* = 5–6, ♦) and P301S mice immunized with Tau_199–208_[pS202/pT205] (*n* = 3‑5, 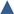), Tau_209–217_[pT212/pS214] (*n* = 5–6, 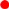), and Tau_229–237_[pT231/pS235] (*n* = 5–6, 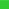) at 32 weeks (upper row) and 48 weeks (lower row) of age obtained from immunoblots. Shown are the relative ratios of phospho-tau (mAbs AT8, AT100, and AT180) to total tau (mAb Tau5) in percent arranged according to the temporal occurrence of the phospho-tau epitopes (pT231/pS235—early, pT212/pS214—intermediate, pS202/pT205—late).

Phosphorylation degrees of the human tau mutant at positions 231/235, 212/214 and 202/205, representing an early, an intermediate and a late phosphorylation event [29,30], were judged in immunoblots after SDS-PAGE by normalizing the relative band intensities obtained with mAbs AT8, AT100, and AT180, respectively, to mAb Tau5 (total tau). It should be noted that the normalized intensities allow only to judge higher or lower phosphorylation degrees of a given epitope (represented by one mAb) among the animal groups, but do not among the three different epitopes. Expectedly, immunoblots of non-transgenic control mice did not display any band in the area where the human tau isoform 412 was detected, whereas transgenic mice displayed intense tau and phospho-tau bands (Figure S2). In general, immunized and non-immunized 48 weeks old mice had higher phosphorylation-degrees than 32-week-old mice on top of the tau expression levels that increased also with age (Figure 5). Although the changes of the phosphorylation degrees among the different groups were not statistically significant, which was attributed to the heterogeneity among the animals of one group and the relatively bad quantification characteristics of immunoblots, there are few interesting trends obvious. MAb AT8 indicated an increasing phosphorylation degree from 32 to 48 weeks in the placebo group, whereas the levels remained stable in the immunized groups and thus were lower than the placebo group, which was even significant for mice immunized with the corresponding epitope Tau_199–208_[pS202/pT205] (one-tailed Mann-Whitney test). A similar but less pronounced trend was visible for mAb AT100, but not for mAb AT180. The latter mAb, however, recognizes also tau mono-phosphorylated at Thr231 and thus does not correctly stain the targeted double phosphorylated tau version.

The phospho-tau to tau ratio increased from 32 weeks (Figure 5, upper panel) to 48 weeks old mice (Figure 5, lower panel) only slightly for mice immunized with Tau_199–208_[pS202/pT205] when analyzed with mAb AT8 (1.3-fold) and AT180 (0.9-fold), whereas mAb AT100 indicated a 8.1-fold increase and lowest increase for mice immunized with Tau_229–237_[pT231/pS235] (1.5-fold). Highest increases of the ratio were detected in placebo treated mice (4.6-fold) when analyzed with mAb AT8 and in Tau_229–237_[pT231/pS235]-immunized mice (2.0-fold) when AT180 was applied (Figure 5, left panel).

### 3.5. Immunohistochemical Quantification of Tau Pathology in P301S Mice

**Figure 6:**
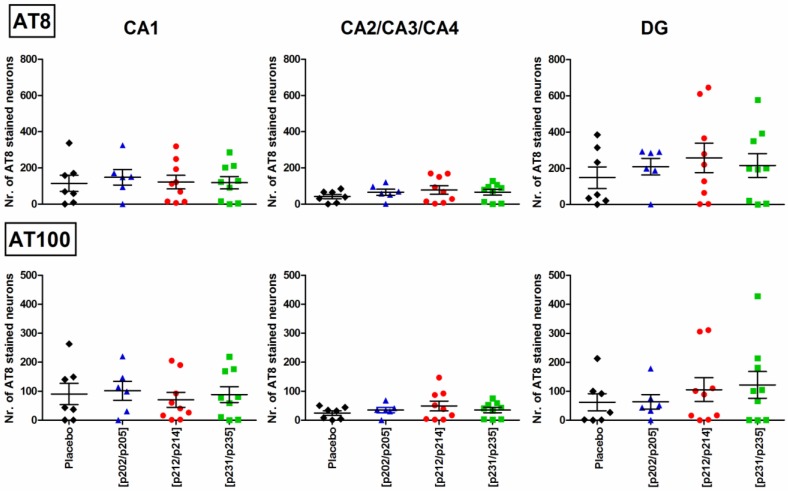
Quantitative immunohistochemistry of tau pathology in 48 weeks old P301S mice. Cells positively stained with mAbs AT8 and AT100 were counted within the pyramidal cell layer of region CA1 (left), CA2/3/4 (middle) and the granular cell layer of DG (right) of P301S mice treated with placebo (*n* = 7, ♦), Tau_199–208_[pS202/pT205] (*n* = 6, 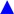), Tau_209–217_[pT212/pS214] (*n* = 9, 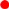), or Tau_229–237_[pT231/pS235] (*n* = 9, 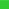).

At coronal brain slices of non-transgenic mice no immunostaining of phosphorylation dependent mAbs AT8 (Tau[pS202/pT205]) and AT100 (Tau[pT212/pS214]) could be found corroborating the lack of pathology in these mice (data not shown). In placebo-treated and vaccinated P301S mice both mAbs AT8 and AT100 stained neurons, which could be quantified (Figure 6). At 48 weeks the phospho-tau pathology was most obvious in the cortex, hippocampus, amygdala, piriform cortex, and hypothalamic region. The microscope images displayed star shaped structures comprised of a dense staining of the entire cell body and protrusions without staining of the nucleus (Figure S1, left). Within the hippocampal formation phospho-tau positive neurons appeared uniformly distributed in the visual field (Figure S1, right). The intensity of these structures increased from 32-week to 48-week-old animals.

The tau pathology was quantitatively evaluated by counting cells positively stained for hyperphosphorylated tau in CA1 and CA2/3/4 regions and the dentate gyrus (DG) (marked zones in Figure S1). The analysis revealed great differences in the manifestation of pathology between the individuals, which was also reflected by immunoblot analysis. In addition, cells positively stained for hyperphosphorylated tau were heterogeneously distributed in the evaluated regions and heterogeneity was also observed for all studied phosphorylation sites in all hippocampal regions (Figure 6). CA1 region and the combined CA2/3/4 regions showed nearly equal mean values among the placebo-treated and the three immunization groups for mAbs AT8 and AT100. Counts of cells positively stained for hyperphosphorylated tau in the CA1 region were between 91 (AT100) to 130 (AT8) and in the combined CA2/3/4 region between 36 (AT100) to 64 (AT8) counts. The DG contained 1.2- to 1.5-fold more AT8 positive stained cells (209 to 258 counts) in the three immunization groups than in the placebo-group (174 counts). The lowest number of mAb AT100 positive cells within the DG was counted for Tau_199–208_[pS202/pT205] immunized mice (55 counts) and around 2.2-fold more in mice immunized with Tau_229–237_[pT231/pS235] (122 counts, Figure 6). The differences, however, were again not statistically significant when comparing the immunized and placebo treated groups.

### 3.6. Behavioral Characterization of P301S Mice

**Figure 7:**
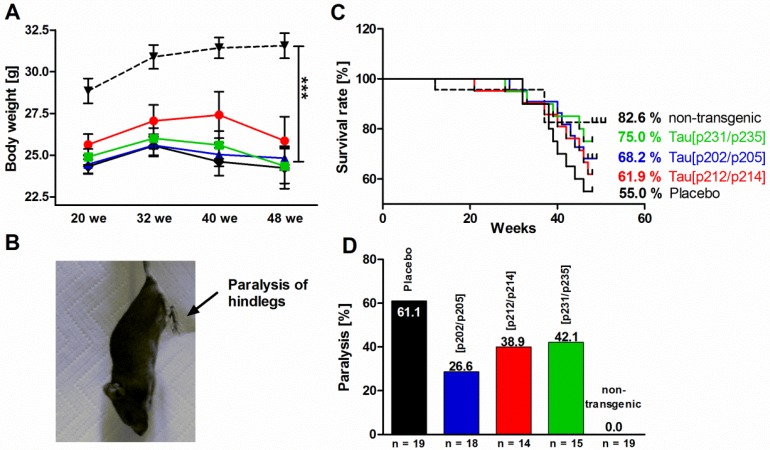
Health monitoring of non-transgenic mice (control, ▼, *n* = 19) and P301S mice treated with a placebo (♦, *n* = 20), Tau_199–208_[pS202/pT205] (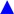, *n* = 15), Tau_209–217_[pT212/pS214] (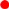, *n* = 16), or Tau_229–237_[pT231/pS235] (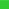, *n* = 20). (**A**) body weight; (**B**) photograph of a paralyzed P301S mouse; (**C**) survival rates; (**D**) percentage of paralysis. Statistical significances are marked by asterisks (***, *p* < 0.001).

The animal behavior was tested with the same group of P301S mice (*n* = 20 per test group) at the age of 20, 32, and 48 weeks. At all ages, non-transgenic mice (control) were significantly, up to 28%, heavier than age-matched transgenic mice (Figure 7A). With an average weight of 25 g there was no significant difference in body weight between vaccinated and placebo-treated transgenic P301S mice. All animals underwent a health monitoring at each point of analysis. The phenotype of clasping and limb retractions was also recorded, but was not statistical significant (data not shown).

Non-transgenic mice had the highest survival rate of 82.6% after 48 weeks, followed by mice vaccinated with Tau_229–237_[pT231/pS235] (75%), Tau_199–208_[pS202/pT205] (68.2%) and Tau_209–217_[pT212/pS214] (61.9%). Only 55% of placebo-treated mice reached this final stage (Figure 7B). Survival analysis by the Log-rank (Mantel-Cox) test and the Log-rank test for trend, however, revealed no statistically significant differences between vaccinated and placebo-treated P301S mice. The p-values for comparing the placebo group with vaccinated groups were 0.19 (Tau_229–237_[pT231/pS235]), 0.35 (Tau_199–208_[pS202/pT205]), and 0.61 (Tau_209–217_[pT212/pS214]). For non-transgenic mice the *p*-values were 0.08 (*vs.* placebo), 0.16 (*vs.* Tau_209–217_[pT212/pS214]), 0.32 (*vs.* Tau_199–208_[pS202/pT205]), and 0.59 (*vs.* Tau_229–237_[pT231/pS235], Figure 7C).

The longer survival rates of vaccinated P301S mice were further supported by the percentage of paralyzed mice (Figure 7D). Whereas non-transgenic mice did not show paralyzed hind limbs and a hunched back posture, these symptoms appeared in 61.1% of the placebo-treated P301S mice at the age of 48 weeks or earlier. At this age only 26.6% to 42.1% of the immunized mice were paralyzed. The life span data shown here are not necessarily linked to the degree of paralysis as we observed paralyzed surviving animals until the end of the experiment.

**Figure 8:**
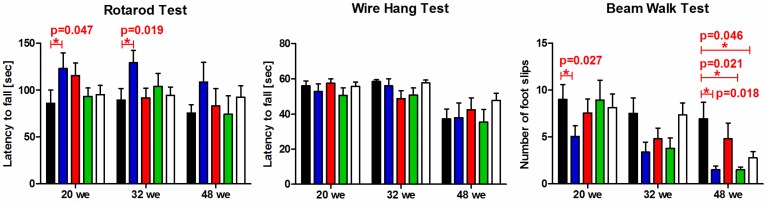
Performance of P301S mice treated with the placebo (black, *n* = 20), immunized with Tau_199–208_[pS202/pT205] (blue, *n* = 15), Tau_209__–217_[pT212/pS214] (red, *n* = 16), or Tau_229–237_[pT231/pS235] (green, *n* = 20), and non-transgenic control mice (white, *n* = 19) in rotarod, wire hang and beam walk tests. Statistical significances are marked by asterisks (*, *p* < 0.05). Given is the age of the mice at which the behavioral test was carried out (we = weeks).

Due to the significant increase of body weight, the performance of non-transgenic mice did not improve over a period of 28 weeks in accelerating rotarod and wire-hang test. At the beam walk test over this time period an improvement of the number of foot slips was observed. Transgenic mice vaccinated with Tau_199–208_[pS202/pT205] showed significantly better motor functions at weeks 20 and 32 in rotarod and beam walk tests than placebo-treated mice, e.g., the latency to fall at the rotarod was in average 123 second for Tau_199–208_[pS202/pT205] vaccinated and only 86 second for placebo-treated mice. Among all three tests, the beam walk revealed a significant better performance of vaccinated animals than compared to placebo-treated mice (Figure 8, right panel). The average number of foot slips at 48 weeks of age for the placebo-treated mice was 7 whereas only 2–5 foot slips were counted for vaccinated mice. Most importantly, this was statistically significant for vaccines Tau_199–208_[pS202/pT205] and Tau_229–237_[pT231/pS235] relative to the placebo group. It should be noted, that the beam walk test is the least challenging test applied here and thus is indicative of already slight improvements of motor capabilities.

Motor deficits increased with age in both placebo-treated and immunized mice, which was most obvious in placebo-treated mice that lost most neuromuscular strength (wire hang test) when comparing their performance at 20 and 48 weeks of age (Figure 8, from 56.0 s to 37.2 s, *p* = 0.007 in Mann Whitney test). Mice immunized with Tau_209–217_[pT212/pS214] showed also a significant reduction form 57.5 s to 42.5 s (*p* = 0,016), whereas for the other two immunization groups no statistically significant decline of strength between the performance at 20 and 48 weeks of age was detected.

Mice were not tested for deficits in spatial learning and memory at the later ages, because application of the Morris water maze at the age of ten months in non-transgenic and transgenic P301S mice showed that they could not be differentiated sufficiently [48].

## 4. Discussion

Alzheimer’s disease is characterized by a massive loss of neurons in the brain that is accompanied by extensive pathological aggregates, *i.e.*, SPs and NFTs containing Aβ_38–43_- and hyperphosphorylated tau versions, respectively. Though it is still unclear if these pathological hallmarks are linked to the onset of the disease or only late events, current data clearly indicate that both deposits contribute to the cognitive deficits of AD and thus should be prevented or reduced to postpone clinical symptoms.

This active immunization study relied on the tau transgenic mouse P301S generated by Yoshiyama *et al.* which show clasping and limb retraction deficits at an age of three month, followed by limb weakness and brain atrophy and develop severe AD-like tangle pathology at an age of seven to ten months [32]. Therefore, these mice are considered as a harsh model for testing the effectiveness of therapeutic inventions. Thus, tau pathology, motor dysfunctions and reduced life-span due to paralysis can be used to evaluate the effectiveness of the vaccines at different levels [32]. It was already shown for this mouse model that anti-tau antibodies can prevent the formation of tau aggregates *in vitro*, can reduce hyperphosphorylated tau recognized by mAb AT8 (pS202/pT205) and can improve cognitive abilities of P301S mice [49,50].

Active Aβ immunization in humans faced the problem of severe side effects caused by pro-inflammatory T cell epitopes within the Aβ sequence, whereas immunizations with tau have only been tested in animals so far [9,51,52,53,54,55]. These approaches however appear to be promising based on different studies either relaying on active [13,14,16,17,56] or passive immunization strategies [15,50,57]. Whereas immunization of female C57BL/6 mice with full-length recombinant tau induced NFT-like pathology and neurological deficits [58], other active and passive immunization studies relied on pathologically altered (e.g., phosphorylated) tau species as immunogens to avoid interference with the physiological tau.

Our study focused on the development of vaccines that contain phosphorylation patterns which are considered to be AD specific to reduce possible adverse effects, like axonal damage and gliosis or the occurrence of tau autoantibodies, as reported by others [13,26,58]. Additionally, the vaccines should provide a strong Th2-polarized immune response with high and long lasting epitope specific titers using an adjuvant approved for humans.

As B cell epitopes we chose three promising presumably Alzheimer’s disease-specific double phosphorylated neoepitopes of the tau protein, namely Tau_199–208_[pS202/pT205], Tau_209–217_[pT212/pS214], and Tau_229–237_[pT231/pS235], which were also used by others but in a different setup [14,26]. As a new advancement these peptides were linked to foreign immune-stimulating T cell epitopes originating either from the tetanus toxin of *Clostridium tetani* or the Ag85B (protein) from *Mycobacterium tuberculosis*. The final vaccines were administered in Alu-GelS (aluminum hydroxide) to stimulate the humoral immune response effectively [27,59,60] rather than coupling to an adjuvant carrier protein, which mainly results in Th1 activation. Compared to recent studies [14,26,52], which rely on adjuvants, such as Freund’s adjuvants, also known for its pro-inflammatory properties [25,26], this strategy may result in a weaker immune response, but aluminum-based adjuvants are allowed for human applications. These depot-forming carriers extent the duration of the B and T cell activation so that a Th2 mediated immune response via inflammasome activation and accumulation of monocytes, GR1^+^ cells, eosinophils and neutrophils emerges at the injection site.

The antigen-specific ELISA confirmed for all peptide vaccines high phospho-tau IgG titers of around 50,000 after the second boost. IgG titers were specific for the respective immunized double phosphorylated epitope and all three epitopes were equally immunogenic in terms of evoking an immune response. Determination of IgG subtypes showed significantly higher IgG_1_ titers compared to IgG_2_ subtypes, which is characteristic for a humoral, antibody based, Th2 polarized immune response. This finding clearly supports the strategy of combining short AD specific epitopes with foreign T cell epitopes to modulate the immune response towards an anti-inflammatory Th2 based immune response. All titers decreased to ~4000 over the following six to seven months indicating that the immune system of the immunized mice was not restimulated, although it is known that endogenous hyperphosphorylated tau protein increases with age in this model [32]. The observed decrease of antibody titer in the blood also results in a decreased antibody concentration in the brain, which presumably contributes to the lack of a sustainable effect. However, assuming that 0.1%–0.2% of the peripheral circulating IgG cross the blood brain barrier and enter the brain or the cerebrospinal fluid these antibodies either need to have extremely high affinities for their target or have to be available in large doses [50]. As no other immunization protocols or vaccine dosages were tested, the reason for this drop and the effects on the antibody concentration in the brain remains unclear.

High phospho-tau specific IgG titers in the early phase of the study (20 weeks) match with the improved behavioral performance in the beam walk and rotarod test for mice immunized with Tau_199–208_[pS202/pT205]. This was not seen for the two other immunization groups although comparable high antibody titers were measured in the early phase. It can be speculated whether the phosphorylation sites Tau[pT231/pS235] and Tau[pT212/pS214], representing early and intermediate phosphorylation events are of lower importance for the development of AD like pathology than Tau[pS202/pT205], a rather late phosphorylation event [29,30]. However, it should also be considered that mice might phosphorylate human tau different than humans. In this respect the positive result obtained for immunization with the peptide vaccine containing the phospho-tau epitope Tau[pS202/pT205] confirmed our vaccination strategy, but the other two vaccines should not be disregarded. Despite low antibody titers in the late phase of the study a tendency to increased survival rates, reduced paralysis accompanied by behavioral improvements, as well as lower phospho-tau to tau ratio after Tau_199–208_[pS202/pT205] treatment was observed, which confirms therapeutic effects on a longer period. Nevertheless, it remains unclear why the biochemical finding of a lower phospho-tau to tau ratio after Tau_199–208_[pS202/pT205] vaccination is not reflected in the corresponding histochemical analysis. Translated to human therapies, it might indicate that early prophylactic vaccinations might delay the disease onset even if the antibody titers fade out earlier.

## 5. Conclusions

The current study confirmed that AD specific neoepitopes can be combined with foreign T cell epitopes to induce fast immune responses with high IgG_1_ titers in the blood, characteristic for a Th2 polarized humoral immune response. The vaccination with three short, double phosphorylated tau sequences specifically detected in human NFT significantly improved the survival rates and paralysis in a P301S mouse model. Additionally, the behavioral tests confirmed a therapeutic effect shortly after vaccination and at later stages, which was especially pronounced the vaccine containing the phospho-tau epitope Tau[pS202/pT205], reflecting a rather late phosphorylation event in AD. Although the therapeutic effects were not clearly supported by immunoblot analysis and immunohistochemistry, all data confirm that the vaccine design appears to be a promising treatment option. For further development the peptide vaccines have to be evaluated in different animal models resembling the characteristics of AD more closely. Furthermore the humoral polarization (Th2) of the induced immune response has to be confirmed by determination of IFNγ and IL-4 after re-stimulation of lymphocytes.

## Supplementary Files

Supplementary File 1
