# Supplementary material for: Doubly Phosphorylated Peptide Vaccines to Protect Transgenic P301S Mice against Alzheimer’s Disease Like Tau Aggregation
Source: Vaccines (Basel). 2014 Jul 29;2(3):601–23. doi: 10.3390/vaccines2030601 (PMC4494214; doi:10.3390/vaccines2030601)
Supplement: Supplementary File 1 [file vaccines-02-00601-s001.pdf]

Vaccines **2014**, *2*, 1-11 manuscripts; doi:10.3390/vaccines20x000x

OPEN ACCESS

vaccines

ISSN 2076-393X

www.mdpi.com/journal/vaccines

Article

Doubly Phosphorylated Peptide Vaccines to Protect Transgenic P301S Mice against Alzheimer's Disease Like Tau Aggregation

Monique Richter [1,2], Agneta Mewes [1,2], Manuela Fritsch [1,2], Ute Krügel [3], Ralf Hoffmann [1,2] and David Singer [1,2,*]

[1] Institute of Bioanalytical Chemistry, Faculty of Chemistry and Mineralogy, Universität Leipzig, Leipzig 04103, Germany

[2] Center for Biotechnology and Biomedicine (BBZ), Universität Leipzig, Leipzig 04103, Germany

[3] Rudolf Boehm Institute for Pharmacology and Toxicology, Universität Leipzig, Leipzig 04107, Germany

Supplementary Files—Content

Figure S1. Illustration of hippocampal regions quantified after immunohistochemistry using phosphorylation-dependent antibodies.

Figure S2. Representative immunoblots of brain homogenates of P301S mice for analysis of total tau and phospho-tau. Total tau was stained by mAb Tau5 and phospho-tau with mAbs AT8, AT100 and AT180.

Table S1. IgG titers of mice immunized with **$Tau_{199–208}$[pS202/pT205]**, (BT = behavioral test, x =no blood sample available, IR = immune response).

Table S2. IgG titers of mice immunized with **$Tau_{209–217}$[pT212/pS214]**, (BT = behavioral test, x =no blood sample available, IR = immune response).

Table S3. IgG titers of mice immunized with **$Tau_{229–237}$[pT231/pS235]**, (BT = behavioral test, x =no blood sample available, IR = immune response).

Table S4. $IgG_{1/2a/2b/2c}$ titers of mice immunized with $Tau_{199–208}$[pS202/pT205].

Table S5. $IgG_{1/2a/2b/2c}$ titers of mice immunized with $Tau_{209–217}$[pT212/pS214].

Table S6. $IgG_{1/2a/2b/2c}$ titers of mice immunized with $Tau_{229–237}$[pT231/pS235].

Figure S1. Illustration of hippocampal regions quantified after immunohistochemistry using phosphorylation-dependent antibodies. Exemplarily shown is the hippocampal formation of an untreated P301S mouse (48 weeks of age) using mAb AT8 (Tau[pS202/pT205]). Within the hippocampal formation, the pyramidal cell layer of region CA1 (blue), CA2/3/4 (black) and the granular cell layer of the dentate gyrus (red, DG) are marked (left). In the indicated areas cells positively stained for phospho-tau were counted as exemplified in the magnified picture (right).

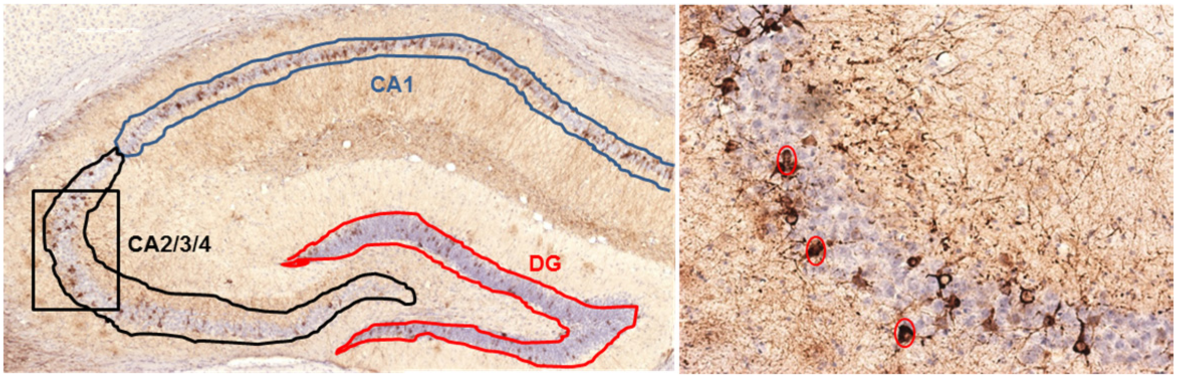

Figure S2. Representative immunoblots of brain homogenates of P301S mice for analysis of total tau and phospho-tau. Total tau was stained by mAb Tau5 and phospho-tau with mAbs AT8, AT100 and AT180. Samples S1–S3: mice immunized with $Tau_{209–217}$[pT212/pS214], S4–S10: mice immunized with $Tau_{229–237}$[pT231/pS235] and S11–S13: placebo-treated P301S mice. As reference (Ref) an untreated P301S mouse was included. Soybean trypsin inhibitor (SBTI) was used as external standard.

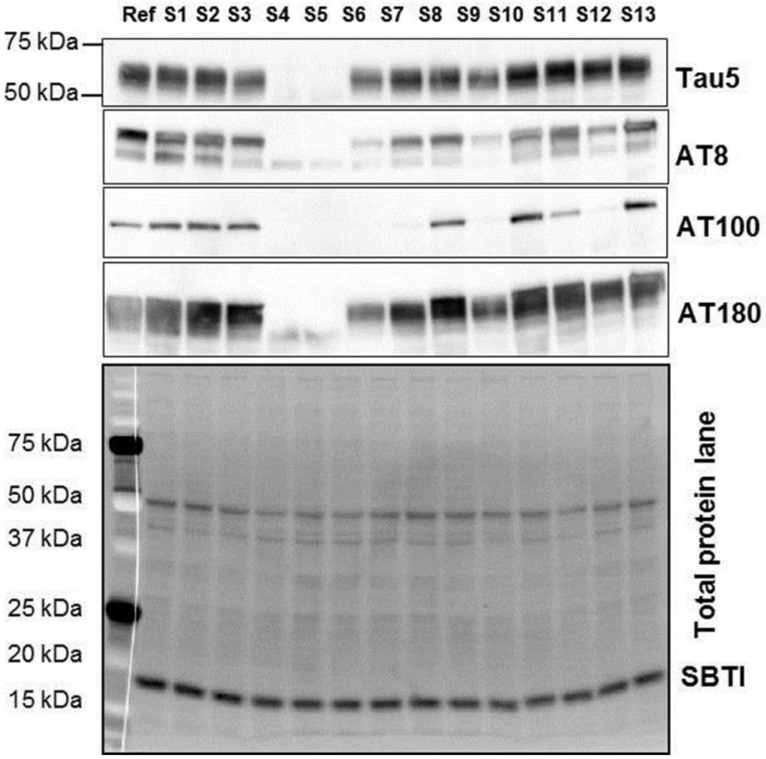

Table S1. IgG titers of mice immunized with **Tau$_{199–208}$[pS202/pT205]**, (BT = behavioral test, x = no blood sample available, IR = immune response).

**Mouse number**	**PreImmune**	**Priming**	**1. Boost**	**2. Boost**	**1 month**	**2 month**	**3 month**	**4/5 month**	**6/7 month**	**final**
**Weeks after immunization**	**0**	**1**	**3**	**7**	**11**	**15**	**19**	**23/27**	**31/35**	**38**
**Age of mice (weeks)**	**10**	**11**	**13**	**27**	**21**	**25**	**29**	**33/37**	**41/45**	**48**
306	0	100	100,000	100,000	100,000	10,000	10,000	1000	1000	1000
309	0	100	100,000	100,000	100,000	10,000	1000	1000	1000	1000
312	0	100	100,000	100,000	100,000	10,000	10,000	1000	1000	100
316	0	10,000	100,000	10,0000	10,000	1000	1000	1000	1000	1000
320	0	1000	100,000	100,000	100,000	10,000	10,000	1000	1000	1000
360	0	1000	100,000	100,000	BT	100,000	10,000	10,000	10,000	10,000
361	0	100	10,000	10,000	BT	10,000	1000	x	x	x
362	0	100	10,000	1000	1000	1000	1000	1000	10000	1000
363	0	1000	10,000	10,000	BT	1000	1000	1000	1000	100
364	0	0	100,000	100,000	BT	100,000	10,000	10,000	10,000	1000
507	0	0	0	0	0	0	0	0	x	0
540	0	0	0	0	100	1000	100	0	0	0
541	0	0	0	0	0	0	0	0	0	0
558	0	100	10,000	1000	1000	0	0	100	1000	100
559	0	0	0	0	0	0	0	0	0	0
627	0	100	10,000	10,000	10,000	100,000	1000	1000	100	1000
628	0	100	10,000	10,000	10,000	10,000	10,000	1000	1000	1000
630	0	1000	100,000	100,000	100,000	100,000	100,000	10,000	10,000	10,000
634	x	100	0	0	0	0	0	0	0	0
626	0	1000	10,000	10,000	1000	10,000	1000			1000
776	0	0	10,000	100,000	100,000	10,000	10,000	10,000		
794	0	0	100,000	100,000	100,000	100,000	10,000	10,000		
812	0	0	100,000	10,000	100,000	10,000	10,000	10,000		
838	0	0	0	100	10000	1000	0	1000		
845	0	0	0	0	0	0	0	0		
850	0	0	10,000	1000	1000	1000	100	1000		

Table S1. *Cont.*

Mouse Number	PreImmune	Priming	1. Boost	2. Boost	1 month	2 month	3 month	4/5 month	6/7 month	Final
**Weeks after Immunization**	**0**	**1**	**3**	**7**	**11**	**15**	**19**	**23/27**	**31/35**	**38**
**Age of Mice (Weeks)**	**10**	**11**	**13**	**27**	**21**	**25**	**29**	**33/37**	**41/45**	**48**
851	0	0	0	0	100	0	0	0		
858	0	0	0	0	0	0	1000	0		
859	0	0	100,000	10,000	100,000	10,000	10,000	10,000		
860	0	0	0	0	0	0	0	0		
876	0	0	10,000	10,0000	10,000	10,000	10,000			
879	0	0	10,000	100,000	10,000	10,000	10,000			
933	0	0	100	1000	1000	1000	100			
1037	0	0	0	0	0	100	0			
1040	0	0	0	0	0	0	0			
1041	0	0	0	0	0	0	0			
1049	0	0	10,000	10,000	10,000	10,000	1000			
1051	0	0	100	100	100	100	100			
1052	0	0	0	0	0	0	0			
1054	0	0	0	0	0	0	0			
**Total number of mice**	**n = 39**	**n = 40**	**n = 40**	**n = 40**	**n = 36**	**n = 40**	**n = 40**	**n = 28**	**n = 17**	**n = 19**
**Total number of mice with IR**	**n = 0**	**n = 15**	**n = 25**	**n = 26**	**n = 24**	**n = 27**	**n = 26**	**n = 19**	**n = 13**	**n = 14**
**[%] of mice with IR**	**0%**	**37.5%**	**62.5%**	**65%**	**66.7%**	**67.5%**	**65%**	**67.8%**	**76.5%**	**73.7%**

Table S2. IgG titers of mice immunized with **Tau$_{209-217}$[pT212/pS214]**, (BT = behavioral test, x = no blood sample available, IR = immune response).

Mouse Number	PreImmune	Priming	1. Boost	2. Boost	1 month	2 month	3 month	4/5 month	6/7 month	Final
**Weeks after Immunization**	**0**	**1**	**3**	**7**	**11**	**15**	**19**	**23/27**	**31/35**	**38**
**Age of Mice (Weeks)**	**10**	**11**	**13**	**27**	**21**	**25**	**29**	**33/37**	**41/45**	**48**
289	0	100	10,000	100,000	10,000	10,000	1000	1000	1000	1000
291	0	100	10,000	100,000	1000	1000	1000	1000	1000	1000
295	100	0	10,000	100,000	10,000	10,000	10,000	1000	100	100
300	0	1000	10,000	10,000	1000	x	x	x	x	x
302	0	0	100,000	100,000	100,000	10,000	10,000	x	x	1000
389	0	0	1000	100	0	0	0	0	0	0
390	0	0	100	10,000	BT	1000	100	100	100	100
391	0	0	1000	1000	1000	100	100	1000	100	100
392	0	0	1000	10,000	BT	10,000	1000	1000	1000	1000
394	0	100	1000	1000	1000	1000	10000	1000	100	100
498	0	0	1000	1000,000	1000,000	10,000	10,000	1000	x	1000
499	0	0	10,000	100,000	10,000	10,000	10,000	1000	10,000	1000
500	0	100	10,000	100,000	100,000	100,00	10,000	10,000	x	1000
537	0	100	100,000	100,000	100,000	10,000	10,000	10,000	1000	10,000
547	0	0	10,000	10,000	10,000	10,000	10,000	10,000	1000	1000
594	0	0	100	1000	x	100	100	0	0	0
596	0	0	0	1000	x	10,000	1000	100	100	100
597	0	100	0	100	x	1000	1000	100	100	100
603	0	100	10,000	1000	1000	1000	1000	100	100	100
605	0	0	0	1000	10,000	1000	10,000	0	1000	100
746	100	100	1000	10,000	10,000	10,000	1000	1000		
748	0	0	10,000	10,000	1000	1000	1000	1000		
753	0	0	1000	1000	1000	1000	100	100		
760	0	0	0	10,000	1000	10,000	1000	1000		
764	0	0	0	100	1000	1000	1000	1000		
765	0	0	100	100	1000	1000	100	100		

Table S2. *Cont.*

Mouse Number	PreImmune	Priming	1. Boost	2. Boost	1 month	2 month	3 month	4/5 month	6/7 month	Final
**Weeks after Immunization**	**0**	**1**	**3**	**7**	**11**	**15**	**19**	**23/27**	**31/35**	**38**
**Age of Mice (Weeks)**	**10**	**11**	**13**	**27**	**21**	**25**	**29**	**33/37**	**41/45**	**48**
766	0	0	1000	1000	1000	10,000	10,000	1000		
813	0	0	100	1000	1000	1000	1000	1000		
814	0	0	1000	1000	1000	1000	100	x		
815	0	0	1000	10,0000	10,0000	10,000	10,000	10,000		
936	0	0	1000	10,000	10,000	1000	1000			
949	0	0	1000	100,000	100,000	10,000	10,000			
951	0	0	1000	100,000	100,000	10,000	100,000			
988	0	0	1000	10,000	10,000	1000	10,000			
1003	0	0	100	10,000	1000	1000	1000			
1004	0	0	100	10,000	10,000	10,000	10,000			
1005	0	0	100	1000	1000	1000	x			
1019	0	0	100	10,000	10,000	1000	1000			
1020	0	0	1000	10,000	10,000	10,000	10,000			
1022	0	0	10,000	10,000	10,000	10,000	10,000			
**Total number of mice**	**n = 40**	**n = 40**	**n = 40**	**n = 40**	**n = 35**	**n = 39**	**n = 38**	**n = 27**	**n = 16**	**n = 19**
**Total number of mice with IR**	**n = 2**	**n = 9**	**n = 35**	**n = 40**	**n = 34**	**n = 38**	**n = 37**	**n = 24**	**n = 14**	**n = 17**
**[%] of mice with IR**	**5%**	**22.5%**	**77.8%**	**100%**	**97.1%**	**97.4%**	**97.4%**	**88.9%**	**87.5%**	**89.5%**

Table S3. IgG titers of mice immunized with **Tau$_{229-237}$[pT231/pS235]**, (BT = behavioral test, x =no blood sample available, IR = immune response).

**Mouse Number**	**PreImmune**	**Priming**	**1. Boost**	**2. Boost**	**1 month**	**2 month**	**3 month**	**4/5 month**	**6/7 month**	**Final**
**Weeks after Immunization**	**0**	**1**	**3**	**7**	**11**	**15**	**19**	**23/27**	**31/35**	**38**
**Age of Mice (Weeks)**	**10**	**11**	**13**	**27**	**21**	**25**	**29**	**33/37**	**41/45**	**48**
275	0	100	100,000	100,000	100,000	10,000	10,000	1000	1000	1000
280	100	100	100,000	100,000	100,000	100,000	10,000	10,000	1000	100
282	0	100	10,000	10,000	10,000	1000	0	1000	1000	1000
283	0	100	10000	10000	10000	1000	1000	100	0	1000
278	0	100	100,000	100,000	100,000	10,000	x	x	x	10,000
375	0	1000	10,000	100,000	BT	10,000	1000	100	100	1000
383	0	1000	1000	10,000	BT	1000	10,000	1000	100	100
424	0	1000	100,000	100,000	BT	100,000	100,000	100,000	10,000	1000
451	0	1000	10,000	10,000	BT	10,000	10,000	10,000	10,000	1000
452	0	100	100,000	100,000	BT	1000	1000	1000	1000	10,000
455	100	100	100,000	100,000	100,000	10,000	10,000	10,000	10,000	10,000
456	100	100	100,000	100,000	100,000	10,000	10,000	10,000	10,000	10,000
481	0	100	100,000	100,000	10,000	10,000	10,000	1000	1000	1000
506	100	100	10,000	100,000	10,000	1000	1000	1000	1000	1000
511	100	0	100,000	100,000	100,000	1000	1000	1000	1000	1000
662	0	1000	100,000	10,000	10,000	10,000	10,000	1000	1000	1000
664	0	0	100	1000	10,000	10,000	1000	1000	100	100
665	0	1000	100,000	100,000	100,000	100,000	100,000	10,000	10,000	10,000
682	0	100	100,000	100,000	100,000	100,000	10,000	x	x	1000
684	x	0	10,000	10,000	10,000	10,000	1000	100	x	100
730	0	0	1000	1000	1000	1000	1000	1000		
735	0	0	1000	10,000	10,000	10,000	1000	100		
737	0	0	1000	10000	10000	1000	1000	1000		
742	0	0	100,000	10,000	10,000	1000	1000	x		
787	0	0	10,000	100,000	10,000	10,000	1000	1000		
789	0	0	100,000	x	x	x	x	x		

Table S3. *Cont.*

Mouse Number	PreImmune	Priming	1. Boost	2. Boost	1 month	2 month	3 month	4/5 month	6/7 month	Final
**Weeks after Immunization**	**0**	**1**	**3**	**7**	**11**	**15**	**19**	**23/27**	**31/35**	**38**
**Age of Mice (Weeks)**	**10**	**11**	**13**	**27**	**21**	**25**	**29**	**33/37**	**41/45**	**48**
790	0	0	100,000	100,000	100,000	10,000	10,000	1000		
798	0	0	100,000	100,000	100,000	100,000	10,000	10,000		
804	0	0	1000	100,000	100,000	100,000	10,000	10,000		
807	0	0	1000	10,000	10,000	1000	1000	1000		
885	0	0	10,000	10,000	10,000	1000	1000			
887	0	0	10,000	10,000	100,000	10,000	10,000			
952	0	0	10,000	10,000	10,000	10,000	1000			
963	0	0	100,000	100,000	100,000	10,000	10,000			
965	0	0	1000	1000	1000	100	100			
973	0	0	1000	10,000	10,000	10,000	1000			
974	0	0	1000	1000	10000	1000	1000			
982	0	0	10,000	10,000	10,000	10,000	1000			
966	x	x	x	x	x	x	x			
1029	0	0	10000	x	x	x	x			
**Total number of mice**	**n = 39**	**n = 39**	**n = 39**	**n = 37**	**n = 32**	**n = 37**	**n = 36**	**n = 26**	**n = 17**	**n = 20**
**Total number of mice with IR**	**n = 5**	**n = 17**	**n = 39**	**n = 37**	**n = 32**	**n = 37**	**n = 35**	**n = 26**	**n = 16**	**n = 20**
**[%] of mice with IR**	**12.8%**	**43.6%**	**100%**	**100%**	**100%**	**100%**	**97.2%**	**100%**	**94.1%**	**100%**

Table S4. $IgG_{1/2a/2b/2c}$ titers of mice immunized with $Tau_{199-208}$[pS202/pT205], mo = months after the 2. boost.

	IgG1				IgG2a				IgG2b				IgG2c			
	$Tau_{199-208}$[pS202/pT205]															
**Mouse Number**	**2. boost**	**2 mo**	**5 mo**	**final**	**2. boost**	**2 mo**	**5 mo**	**final**	**2. boost**	**2 mo**	**5 mo**	**final**	**2. boost**	**2 mo**	**5 mo**	**final**
306	10,000	10,000	1000	1000	1000	100	0	0	10,000	10,000	1000	100	10,000	1000	1000	100
309	100,000	10,000	100	1000	1000	100	0	100	100	0	0	100	0	0	0	0
312	100,000	100,00	1000	100	0	0	0	0	1000	100	100	0	100	0	0	0
316	100,000	1000	100	100	100	0	0	0	1000	100	1000	100	1000	100	1000	100
320	10,000	10,000	1000	1000	0	0	0	0	1000	1000	1000	100	100	0	0	0
360	100,000	100,000	10,000	10,000	10,000	10,000	1000	1000	1000	100	100	100	100	0	0	0
362	1000	1000	100	100	0	0	0	0	0	0	100	0	0	0	0	0
363	10,000	1000	1000	100	0	0	0	0	100	100	100	0	100	100	100	0
364	100,000	10,000	1000	1000	100	100	0	0	100	100	1000	0	100	1000	100	0
558	1000	0	0	0	0	0	0	0	0	0	0	0	0		0	0
627	10,000	1000	100	0	100	0	0	0	10,000	1000	100	100	1000	100	100	100
628	10,000	10,000	1000	100	0	0	0	0	100	1000	100	100	100	100	100	0
**mean value**	**46000**	**13667**	**1367**	**1208**	**1025**	**858**	**83**	**92**	**2033**	**1125**	**383**	**58**	**1050**	**218**	**200**	**25**

Table S5. $IgG_{1/2a/2b/2c}$ titers of mice immunized with $Tau_{209-217}$[pT212/pS214], mo = months after the 2. boost.

	**IgG1**				**IgG2a**				**IgG2b**				**IgG2c**			
	$Tau_{209-217}$[pT212/pS214]															
**Mouse Number**	**2. boost**	**2 mo**	**5 mo**	**final**	**2. boost**	**2 mo**	**5 mo**	**final**	**2. boost**	**2 mo**	**5 mo**	**final**	**2. boost**	**2 mo**	**5 mo**	**Final**
**#289**	10,000	1000	100	100	100	100	0	0	1000	1000	1000	100	100	1000	100	100
**#291**	10,000	0	100	0	100	0	100	100	1000	100	100	100	1000	0	100	0
**#295**	100,000	10,000	1000		100	100	0		1000	1000	100	0	1000	1000	1000	100
**#390**	10,000	1000	0	0	1000	100	0	0	100	0	0	0	0	0	0	0
**#391**	100	100	1000	0	0	0	100	0	100	0	0	0	0	0	0	0
**#392**	10,000	10,000	1000	100	1000	1000	1000	100	100	0	0	0	0	0	0	0
**#394**	1000	1000	100	0	0	100	0	0	0	100	0	0	0	100	100	0
**#498**	100,000	10,000	1000	1000	10,000	10,000	1000	1000	1000	100	100	100	100	0	0	0
**#499**	10,000	1000	100	100	1000	100	0	0	10,000	1000	1000	1000	10,000	1000	1000	100
**#500**	100,000	10,000	1000	1000	0	100	0	0	100	100	100	0	0	0	0	0
**#537**	100,000	10,000	10,000	1000	0	0	0	0	1000	1000	1000	100	100	100	100	0
**#547**	10,000	10,000	1000	1000	0	0	0	0	1000	0	1000	100	100	0	0	0
**mean value**	**38425**	**5342**	**1367**	**391**	**1108**	**967**	**183**	**109**	**1367**	**367**	**367**	**125**	**1033**	**267**	**200**	**25**

Table S6. $IgG_{1/2a/2b/2c}$ titers of mice immunized with $Tau_{229-237}$[pT231/pS235], mo = months after the 2. boost.

	**IgG1**				**IgG2a**				**IgG2b**				**IgG2c**			
	$Tau_{229-237}$[pT231/pS235]															
**Mouse Number**	**2. boost**	**2 mo**	**5 mo**	**final**	**2. boost**	**2 mo**	**5 mo**	**final**	**2. boost**	**2 mo**	**5 mo**	**final**	**2. boost**	**2 mo**	**5 mo**	**Final**
**#275**	10,000	10,000	1000	100	0	0	0	0	1000	1000	100	100	100	0	0	0
**#282**	10,000	1000	100	0	1000	100	0	0	100	0	0	0	100	0	0	0
**#375**	100,000	10,000	1000	100	1000	100	0	0	1000	100	100	0	100	100	0	0
**#383**	10,000	100	100	0	100	0	0	0	100	100	0	0	100	0	0	0
**#424**	100,000	100,000	10,000	1000	100	100	0	0	10,000	1000	1000	1000	10,000	1000	1000	100
**#451**	10,000	10,000	1000	0	0	100	0	0	1000	0	0	100	100	0	100	100
**#452**	100,000	1000	100	1000	1000	0	0	0	10,000	0	0	1000	10000	0	0	100
**#455**	100,000	0	10,000	1000	0	0	100	0	1000	1000	100	100	100	1000	1000	100
**#456**	100,000	1000	1000	100	1000	100	100	0	100,000	10,000	10,000	1000	100,000	10,000	10,000	1000
**#481**	10,000	10,000	100	1000	0	100	0	0	1000	10,000	100	100	1000	1000	100	0
**#506**	10,000	1000	1000	100	0	0	0	100	100	0	0	0	100	0	0	0
**#511**	100,000	1000	1000	100	100	0	0	0	10,000	0	1000	100	10,000	0	100	0
**mean value**	**55000**	**12092**	**2200**	**375**	**358**	**50**	**17**	**8**	**11275**	**1933**	**1033**	**292**	**10975**	**1092**	**1025**	**117**
